# Targeting IRE1α-JNK-c-Jun/AP-1-sEH Signaling Pathway Improves Myocardial and Coronary Endothelial Function Following Global Myocardial Ischemia/Reperfusion

**DOI:** 10.7150/ijms.74533

**Published:** 2022-08-15

**Authors:** Hong-Mei Xue, Wen-Tao Sun, Huan-Xin Chen, Guo-Wei He, Qin Yang

**Affiliations:** 1 The Institute of Cardiovascular Diseases & Department of Cardiovascular Surgery, TEDA International Cardiovascular Hospital, Chinese Academy of Medical Sciences and Peking Union Medical College & Tianjin University, Tianjin, China.; 2 Department of Physiology, Hebei Medical University, Shijiazhuang, Hebei, China.; 3 University of Health and Rehabilitation Sciences, Qingdao, Shandong, China.; 4 Drug Research and Development Center, Wannan Medical College, Wuhu, Anhui, China.; 5 Department of Surgery, Oregon Health and Science University, Portland, Oregon, USA.

**Keywords:** Cardiac function, Endoplasmic reticulum stress, Endothelial function, Ischemia/Reperfusion injury, Soluble epoxide hydrolase

## Abstract

**Objectives:** Endoplasmic reticulum (ER) stress and soluble epoxide hydrolase (sEH) upregulation/activation have been implicated in myocardial ischemia/reperfusion (I/R) injury. We previously reported that ER stress mediates angiotensin II-induced sEH upregulation in coronary endothelium, whether and how ER stress regulates sEH expression to affect postischemic cardiac function remain unexplored. This study aimed to unravel the signaling linkage between ER stress and sEH in an *ex vivo* model of myocardial I/R injury.

**Methods:** Hearts from male Wistar-Kyoto rats were mounted on a Langendorff apparatus and randomly allocated to 7 groups, including control, I/R (30-min ischemia and 60-min reperfusion), and I/R groups pretreated with one of the following inhibitors: 4-PBA (targeting: ER stress), GSK2850163 (IRE1α), SP600125 (JNK), SR11302 (AP-1), and DCU (sEH). The inhibitor was administered for 15 min before ischemia with a peristaltic pump. Hemodynamic parameters including left ventricular systolic pressure (LVSP), left ventricular end-diastolic pressure (LVEDP), and maximal velocity of contraction (+dp/dt_max_) and relaxation (-dp/dt_max_) of the left ventricle were continuously recorded using an intraventricular balloon. Endothelial dilator function of the left anterior descending artery was studied in a wire myograph upon completion of reperfusion. The expression of ER stress molecules, JNK, c-Jun, and sEH was determined by western-blot.

**Results:** I/R decreased LVSP (105.5±6.4 vs. 146.9±13.4 mmHg), and increased LVEDP (71.4±3.0 vs. 6.0±2.7 mmHg), with a resultant decreased LVDP (34.1±9.2 vs. 140.9±13.1 mmHg). I/R attenuated +dp/dt_max_ (651.7±142.1 vs. 2806.6±480.6 mmHg/s) and -dp/dt_max_ (-580.0±109.6 vs. -2118.0±244.9 mmHg/s) (all ps<0.001). The I/R-induced cardiac dysfunction could be alleviated by 4-PBA (LVSP 119.5±15.6 mmHg, p<0.01; LVEDP 21.2±4.2 mmHg, LVDP 98.3±12.0 mmHg, +dp/dt_max_ 2166.7±208.4 mmHg/s, and -dp/dt_max_ -1350.9±99.8 mmHg/s, all ps<0.001), GSK2850163 (LVSP 113.4±10.9 mmHg, p<0.01; LVEDP 37.1±3.1 mmHg, LVDP 76.3±13.9 mmHg, +dp/dt_max_ 1586.5±263.3 mmHg/s, -dp/dt_max_ -1127.7±159.9 mmHg/s, all ps<0.001), SP600125 (LVSP 113.9±5.6 mmHg, LVDP 40.5±3.3 mmHg, +dp/dt_max_ 970.1±89.8 mmHg/s, all ps<0.01), SR11302 (LVSP 97.9±7.5 mmHg, p<0.01; LVEDP 52.7±8.6mmHg, p<0.001; LVDP 45.2±9.8mmHg, p<0.05; +dp/dt_max_ 1231.5±196.6 mmHg/s, p<0.01; -dp/dt_max_ -658.3±68.9 mmHg/s, p<0.05), or DCU (LVSP 109.9±4.1 mmHg, p<0.01; LVEDP 11.7±1.8 mmHg, LVDP 98.2±4.9 mmHg, +dp/dt_max_ 1869.8±121.9 mmHg/s, and -dp/dt_max_ -1492.3±30.8 mmHg/s, all ps<0.001). The relaxant response of the coronary artery to acetylcholine was decreased after I/R in terms of both magnitude and sensitivity (p<0.001). All inhibitors improved acetylcholine-induced relaxation. Global I/R increased sEH expression and induced ER stress in both myocardium and coronary artery. Inhibition of ER stress or IRE1α downregulated I/R-induced sEH expression and inhibited JNK and c-Jun phosphorylation. Both JNK and AP-1 inhibitors lowered sEH level in myocardium and coronary artery in I/R-injured hearts.

**Conclusions:** This study deciphered the molecular linkage between ER stress and sEH regulation in global I/R insult by uncovering a novel signaling axis of IRE1α-JNK-c-Jun/AP-1-sEH, which provided basis for future research on the therapeutic potential of targeting the IRE1α-JNK-c-Jun/AP-1-sEH axis for ischemic myocardial injury.

## Introduction

Reperfusion injury remains an unsolved clinical problem following reconstruction of regional myocardial blood flow in ischemic heart disease and following cardiac surgical procedures involving cardiopulmonary bypass. Continuous efforts are needed to deepen the understanding of the basic mechanisms upon which new therapeutic strategies may be based to minimize ischemia/reperfusion (I/R) injury.

Disruption of endoplasmic reticulum (ER) homeostasis by stimuli such as I/R may result in the accumulation of unfolded/misfolded proteins and eventually ER dysfunction, collectively known as ER stress, which is sensed by the three ER-transmembrane proteins namely protein kinase R-like ER kinase (PERK), activating transcription factor 6 (ATF6), and inositol-requiring enzyme 1 (IRE1) 1, 2. Disassociation of the ER chaperone glucose-regulated protein 78 (GRP78) from PERK, ATF6, and IRE1 initiates the activation of these proximal effectors of the unfolded protein reaction (UPR). Accumulating evidence obtained from both *in vivo* and *in vitro* studies has suggested the role of ER stress in myocardial I/R injury, showing the role of PERK, ATF6, IRE1, and their downstream molecules such as eukaryotic translation initiation factor 2α (eIF2α), activating transcription factor 4 (ATF4), C/EBP homologous protein, and X‑box binding protein 1 (XBP1) in Ca^2+^ overload, apoptosis, inflammation, and autophagy 2-4.

Soluble epoxide hydrolase (sEH) degrades the cardioprotective epoxyeicosatrienoic acids (EETs) to physiologically inactive dihydroxyeicosatrienoic acids 5-7. sEH upregulation/activation has been implicated in I/R injury. For example, inactivation of sEH with pharmacological inhibitors or by gene deletion reduced infarct size after regional myocardial I/R injury in mice 8, and attenuated cardiac damage and endothelial dysfunction in rat hearts subjected to *ex vivo* I/R 9. Post-translational modification of sEH by S-nitrosation increases the enzyme activity, which was observed in rat I/R model and cell reoxygenation injury model 10. It remains far less well understood how sEH is transcriptionally upregulated in pathological states, though studies in vascular endothelium uncovered a transcriptional factor activator protein-1 (AP-1)-binding motif on the sEH promoter and c-Jun binding to the AP-1 site in angiotensin II-induced upregulation of sEH^11^. We previously reported that ER stress mediates angiotensin II-induced sEH upregulation in coronary endothelium 12. Whether and how ER stress regulates sEH expression to affect postischemic cardiac function remain uninvestigated.

c-Jun N-terminal kinase (JNK) is activated in cardiac I/R injury. Excessive reactive oxygen species and nitric oxide eruption during I/R cause phosphorylation and nucleus translocation of JNK, which induces expression of multiple inflammation and apoptosis-related genes by phosphorylating and activating c-Jun/AP-1 13. The coupling of ER stress to JNK activation was deciphered by the finding of JNK activation by IRE1 14. Activation of the IRE1/JNK pathway mediates the secretion of inflammatory cytokines in tubular epithelial cells subjected to hypoxia-reoxygenation 15 and suppression of IRE1/JNK signaling contributes to myocardial protection conferred by low-dose lipopolysaccharide during I/R 16.

**Figure 1:**
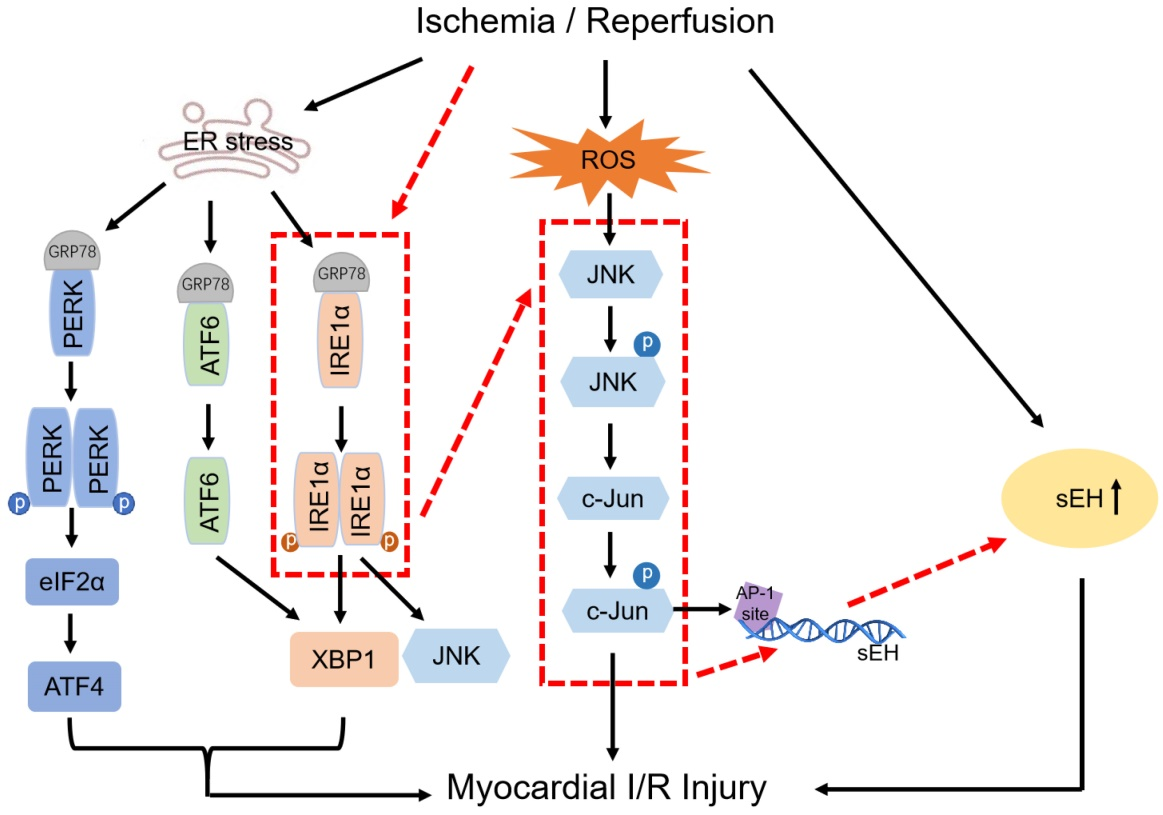
** Illustration of the rationale behind the signaling pathway proposed in this study.** Black solid lines indicate the relationship among factors that have been established in previous studies. Red dotted lines visually show the putative signaling pathway explored in this study.

In this study, by using an isolated rat heart model to mimic the global myocardial I/R encountered in cardiac surgery, we explored the link between ER stress and sEH by testing the hypothesis that IRE1 induces sEH expression via activation of JNK and c-Jun. The putative signaling pathway was proposed (red dotted line) on the basis of the relationship established among factors in previous studies (black solid line), which was illustrated in **Figure 1.** Myocardial function and coronary endothelial function were studied in isolated ischemic/reperfused rat hearts pharmacologically treated with different inhibitors to reveal the potential of targeting IRE1-JNK-c-Jun/AP-1-sEH signaling axis for cardioprotection. In order to evaluate the protective efficacy of the inhibitors per se against global myocardial I/R injury, we did not introduce cardioplegia procedure in this study to avoid complicating protection mechanisms. With the results derived from the present study, further verification of the putative signaling pathway in cardioplegic arrest model that resembles clinical scenario of cardiac surgery seems intriguing.

## Methods

### Animals

Male Wistar-Kyoto rats (300-350g, 8-10 weeks old) purchased from Vital River Laboratory Animal Technology Co. Ltd (Beijing, China) were housed in a humidity- and temperature-controlled environment on a 12h light-dark cycle with free access to food and water before the experiment. The study was approved by the Institutional Ethics Committee of TEDA International Cardiovascular Hospital [TICH-JY-20190125-2] and conformed to the *Guide for the care and Use of Laboratory Animals* published by the US National Institutes of Health (NIH Publication No. 85-23, revised 1985).

### *Ex vivo* model of global myocardial I/R

Rats were anesthetized with 2% sodium pentobarbital (50 mg kg^-1^ *i.p.*). The heart was quickly excised and immediately immersed into 4°C Krebs-Henseleit buffer (KHB). The heart was then mounted on a Langendorff apparatus and perfused with 37°C KHB at a constant hydrostatic pressure of 70-80 mmHg. A water-filled balloon was inserted into the left ventricle and the end-diastolic pressure set to 4-6 mmHg for continuous pressure measurement. Hemodynamic parameters including left ventricular systolic pressure (LVSP), left ventricular end-diastolic pressure (LVEDP), left ventricular developed pressure (LVDP, calculated as LVSP-LVEDP), and maximal velocity of contraction (+dp/dt_max_) and relaxation (-dp/dt_max_) were monitored continuously by a pressure transducer connected to a Powerlab system (AD Instrument, Australia) at a sampling rate of 500 Hz. The heart was perfused for 30-min with warm KHB for stabilization, followed by 30-min global ischemia and 60-min reperfusion. Different pharmacological inhibitors were administered for 15 min before ischemia with a peristaltic pump. Upon completion of reperfusion, the coronary artery was dissected out for isometric force study and the cardiac tissue was snap frozen in liquid nitrogen and stored at -80°C for western blotting analysis.

### Experimental protocols

Fifty-six rats were randomly allocated into 7 groups with 8 rats in each group. The protocol for the Langendorff-perfused heart experiment was as follows and schematically illustrated in ***Figure S1***.

**1) Control group**: Rat hearts were perfused with warm KHB throughout the whole experiment.

**2) I/R group**: Rat hearts were stabilized with warm KHB perfusion for 30 min, then KHB was administrated with a peristaltic pump for 15 min, followed by 30-min ischemia and 60-min reperfusion.

**3)** 4-PBA+I/R group

**4)** GSK2850163+I/R group

**5)** SP600125+I/R group

**6)** SR11302+I/R group

**7)** DCU+I/R group.

In groups 3-7, rat hearts were stabilized for 30 min with KHB, then one of the following inhibitors including ER stress inhibitor 4-phenylbutyric acid (4-PBA, 2 mmol/L) 17, IRE1α inhibitor GSK2850163 (10 μmol/L) 18, c-Jun/JNK inhibitor SP600125 (25 μmol/L) 19, 20, AP-1 inhibitor SR11302 (1 μmol/L) 21, 22, and sEH inhibitor N,N'-Dicyclohexylurea (DCU, 10 μmol/L) 23, 24 was administrated with a peristaltic pump for 15 min before ischemia at a rate of 6 ml/min. During the drug administration, the normal heart perfusion was discontinued.

### Isometric force study of coronary arteries

The left anterior descending artery was cut into rings (2 mm in length) and mounted on a wire myograph (Model 620M, J.P.Trading, Aarhus, Denmark). The rings were equilibrated at 37°C for 30-min, then normalized to obtain an optimal pretension force by setting them to an internal circumference equivalent to a 90% of the circumference at a passive transmural pressure of 100 mmHg 25, 26. Endothelium-dependent relaxation was determined in the rings precontracted with phenylephrine by cumulatively adding acetylcholine (ACh, 10^-9^~10^-5^ mol/L) in the myograph chamber.

To ensure the comparability of the relaxant response among groups, similar extent of precontraction was achieved by using varied concentration of phenylephrine ranging from -7 to -6 LogM.

### Western blotting

Protein extracts from cardiac tissue and coronary artery were used for protein expression analysis of PERK, phosphorylated (Thr980) PERK (p-PERK), IRE1α, phosphorylated (Ser724) IRE1α (p-IRE1α), ATF6, GRP78, JNK, phosphorylated (Thr183+Tyr185) JNK (p-JNK1+JNK2), c-Jun, phosphorylated (Ser63 and Ser73) c-Jun (p-c-Jun), and sEH. Details were described in ***Supplementary Methods***.

### Statistical analysis

All data were expressed as mean±SEM. Analysis and plotting were performed using SPSS (version 20; IBM-SPSS Inc, Armonk, NY) and Graphad Prism8 (San Diego, US). Differences among groups were analyzed by one-way ANOVA followed by Bonferroni's post hoc test and two-way repeated measures ANOVA when appropriate. P<0.05 was considered statistically significant.

## Results

### Inhibition of ER stress ameliorated myocardial systolic and diastolic dysfunction following global myocardial I/R

**Figure 2:**
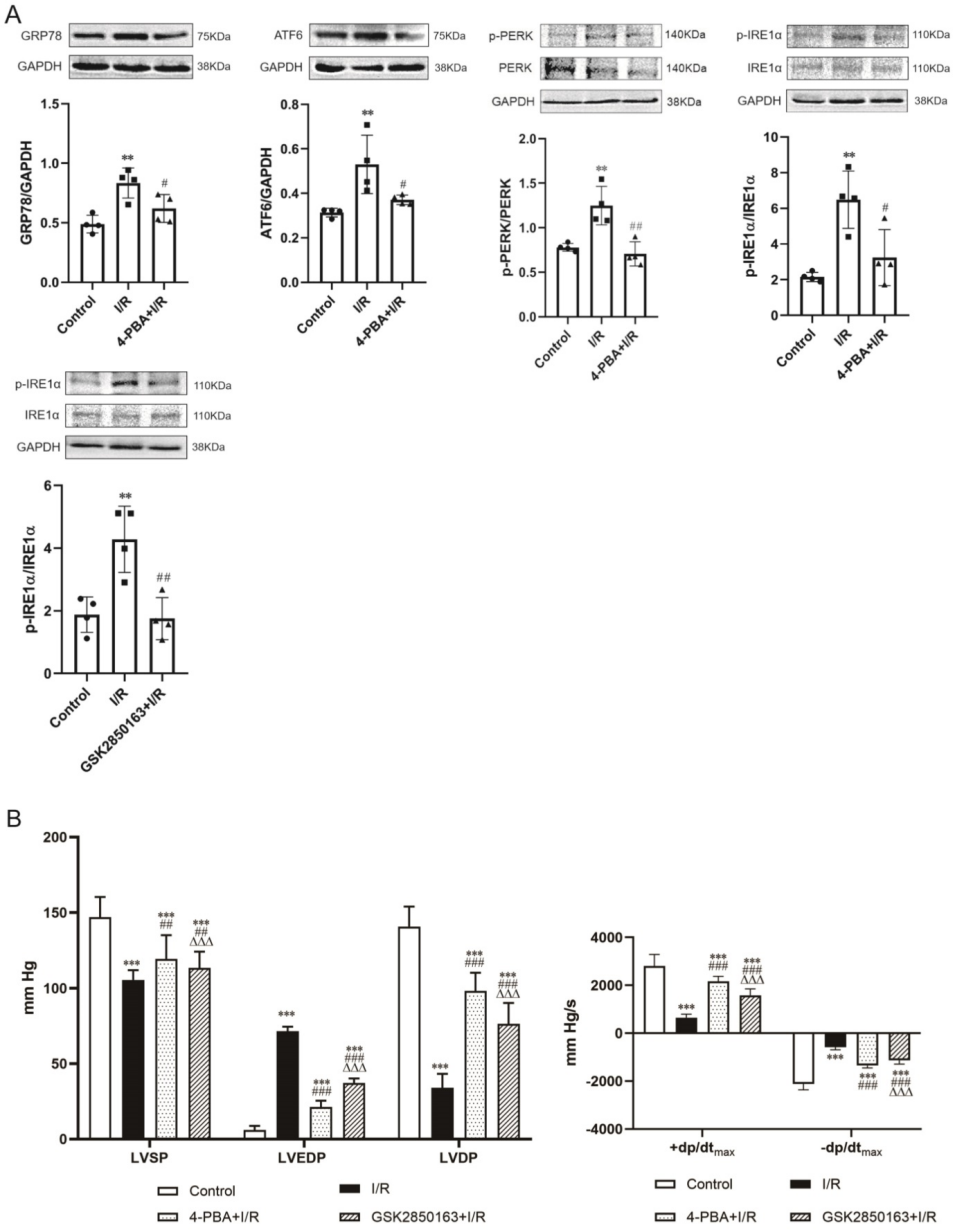
** Activation of the IRE1α branch of ER stress contributes to global ischemia/reperfusion (I/R)-induced myocardial dysfunction. (A)** Western-blot analysis showing the upregulation/activation of ER stress molecules by I/R in myocardium and the effectiveness of 4-PBA in inhibiting I/R-induced ER stress. The IRE1α specific inhibitor GSK2850163 effectively suppressed the activation of IRE1α, evidenced as decreased IRE1α phosphorylation (n=4). **(B)** Functional studies showing the protection conferred by 4-PBA and GSK2850163 on postischemic contractile and diastolic function of the heart (n=8). ^**^p<0.01, ^***^p<0.001 *vs.* Control; ^#^p<0.05, ^##^p<0.01, ^###^p<0.001* vs.* I/R; ^ΔΔΔ^p<0.001 *vs.* 4-PBA+I/R.

The expression of ER stress molecules in the myocardium including GRP78, ATF6, and phosphorylated PERK and IRE1α was significantly increased after 30-min ischemia and 60-min reperfusion, suggesting the induction of ER stress by I/R. Pretreatment with the ER stress inhibitor 4-PBA effectively suppressed I/R-induced upregulation of the ER stress molecules **(*Figure 2A upper panel*)**. The inhibition of ER stress was accompanied by an amelioration of postischemic systolic and diastolic dysfunction of the heart. Compared with the heart subjected to I/R, heart perfused with 4-PBA before I/R showed increases in LVSP (119.5±15.6 vs. 105.5±6.4 mmHg, p<0.01), +dp/dt_max_ (2166.7±208.4 vs. 651.7±142.1 mmHg/s, p<0.001), and -dp/dt_max_ (-1350.9±99.8 vs. -580.0±109.6 mmHg/s, p<0.001) while decreases in LVEDP (21.2±4.2 vs. 71.4±3.0 mmHg, p<0.001). The elevation of LVSP and reduction of LVEDP resulted in an increased LVDP (98.3±12.0 vs. 34.1±9.2 mmHg, p<0.001) **(*Figure 2B*)**. The cardioprotective effect against I/R injury was also observed in the heart perfused with GSK2850163, a novel inhibitor of IRE1α. GSK2850163 improved LVSP (113.4±10.9 mmHg, p<0.01 vs. I/R), LVEDP (37.1±3.1mmHg, p<0.001 vs. I/R), LVDP (76.3±13.9 mmHg, p<0.001 vs. I/R), as well as +dp/dt_max_ (1586.5±263.3 mmHg/s, p<0.001 vs. I/R) and -dp/dt_max_ (-1127.7±159.9 mmHg/s, p<0.001 vs. I/R), which was associated with an inhibition of IRE1α phosphorylation **(*Figure 2A lower panel & Figure 2B*)**. However, GSK2850163 was less effective than 4-PBA in restoration of the systolic and diastolic function of the I/R-injured heart (p<0.001).

### Role of JNK and c-Jun/AP-1 in global I/R-induced cardiac dysfunction: activation by IRE1α branch of ER stress

**Figure 3:**
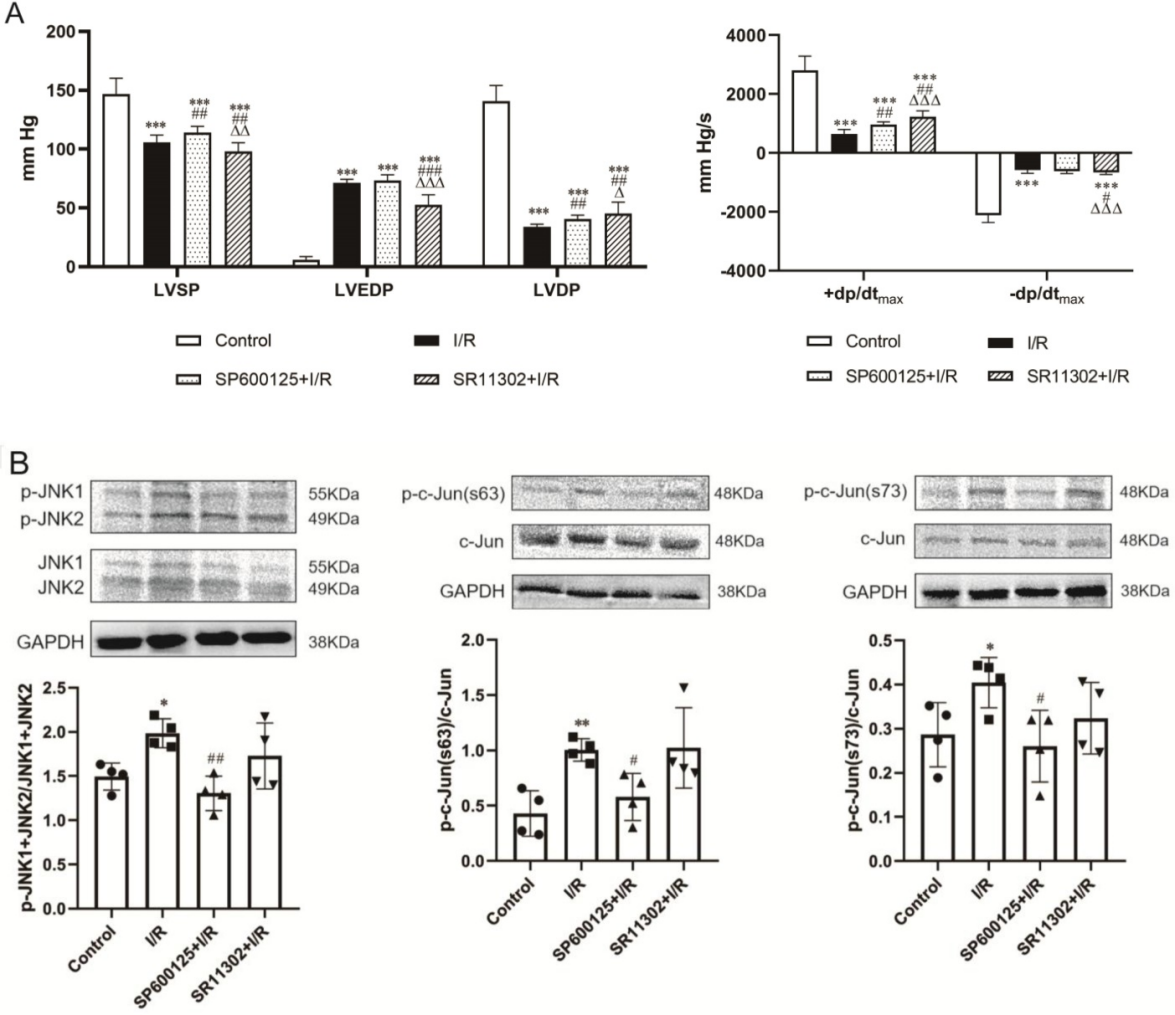

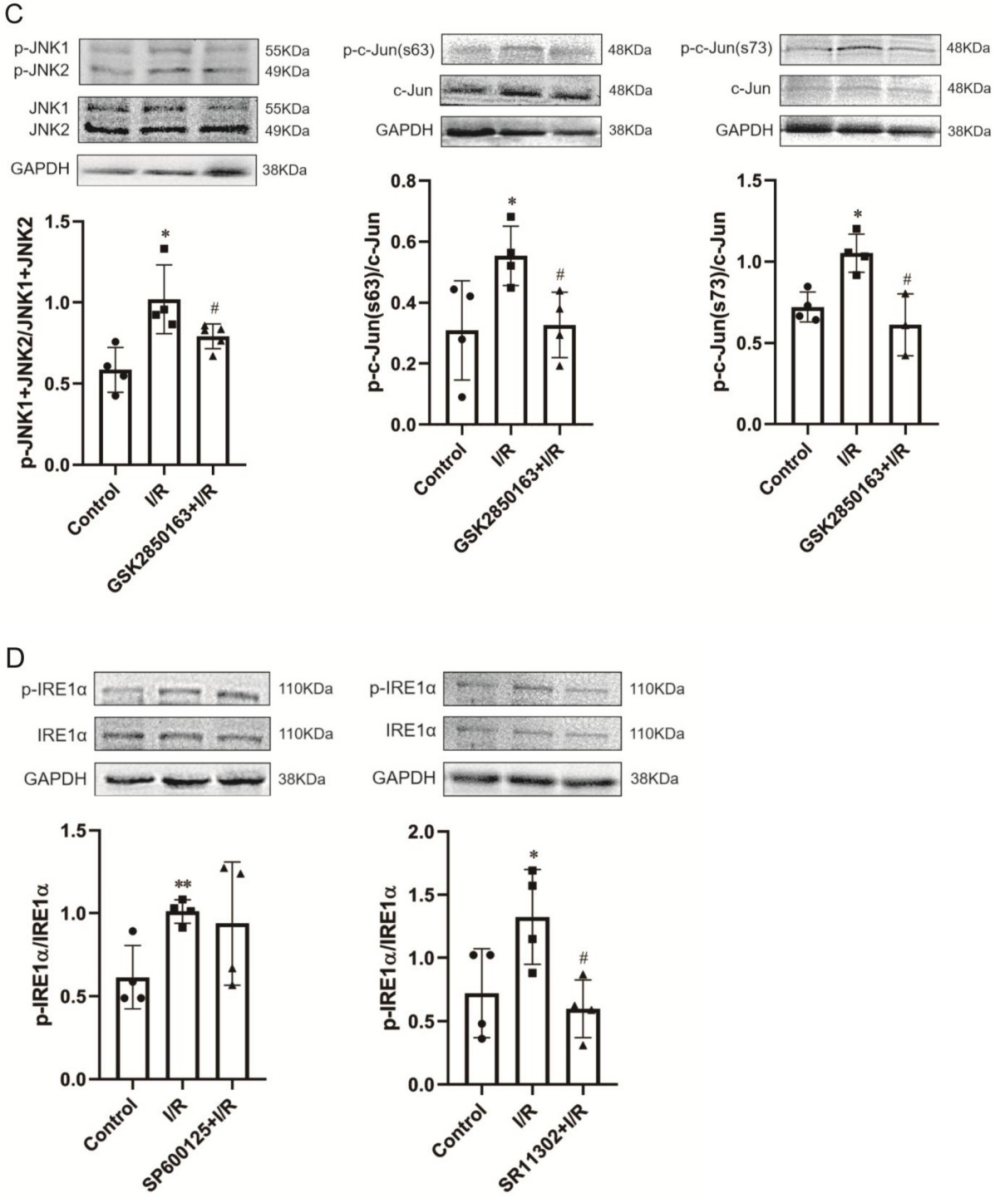
** IRE1α mediates ischemia/reperfusion (I/R)-induced myocardial dysfunction through activation of JNK-AP-1 pathway. (A)** Perfusion of the rat heart with the JNK inhibitor SP600125 and the AP-1 inhibitor SR11302 prior to I/R improved postischemic myocardial function (n=8). **(B)** JNK inhibitor decreased JNK and c-Jun phosphorylation in I/R-injured heart (n=4). **(C)** I/R-induced phosphorylation of JNK and c-Jun in myocardium was suppressed by the IRE1α inhibitor GSK2850163 (n=4). **(D)** Inhibition of AP-1 attenuated the phosphorylation of IRE1α in I/R-injured heart (n=4). ^*^p<0.05, ^**^p<0.01, ^***^p<0.001 *vs.* Control; ^#^p<0.05, ^##^p<0.01, ^###^p<0.001 *vs.* I/R; ^Δ^p<0.05, ^ΔΔ^p<0.01, ^ΔΔΔ^p<0.001 *vs.* SP600125+I/R.

Perfusion of the heart before the onset of ischemia with the selective JNK inhibitor SP600125 or the AP-1 inhibitor SR11302 ameliorated postischemic cardiac dysfunction. SP600125 increased LVSP (113.9±5.6 vs*.* 105.5±6.4 mmHg, p<0.01) though barely improved LVEDP (73.4±4.9 vs. 71.4±3.0 mmHg, p>0.05) in hearts subjected to I/R. In contrast, SR11302-treated hearts showed no improvement in LVSP, which was even lower than in the I/R hearts (97.9±7.5 vs*.* 105.5±6.4 mmHg, p<0.01), whereas the LVEDP was significantly reduced (52.7±8.6 vs. 71.4±3.0 mmHg, p<0.001). Both SP600125 and SR11302 significantly increased LVDP (SP600125: 40.5±3.3 and SR11302: 45.2±9.8 mmHg, p<0.01 vs. 34.1±9.2 mmHg in I/R group) and +dp/dt_max_ (SP600125: 970.1±89.8 *vs.* 651.7±142.1 mmHg/s, p<0.01; SR11302: 1231.5±196.6 *vs.* 651.7±142.1 mmHg/s, p<0.01). SR11302 also ameliorated the reduction in -dp/dt_max_ in postischemic hearts (-658.3±68.9 vs*.* -580.0±109.6 mmHg/s, p<0.05) **(*Figure 3A*)**.

The protein expression of phosphorylated c-Jun (p-c-Jun Ser63 and Ser73) and JNK (p-JNK1/2) were significantly upregulated in rat hearts after 30-min ischemia and 60-min reperfusion, indicating the activation of JNK and c-Jun by I/R. SP600125 inhibited the phosphorylation of JNK and c-Jun whereas SR11302 showed no obvious effect **(*Figure 3B*)**. The I/R-induced enhancement of JNK and c-Jun phosphorylation was observed to be significantly suppressed in hearts pretreated with the IRE1α inhibitor GSK2850163 **(*Figure 3C*)**. Conversely, the phosphorylation of IRE1α by I/R was not affected by SP600125 treatment **(*Figure 3D left panel*)**. Taken together, these results suggested that JNK acts as a downstream molecule of IRE1α activation. The inhibition of IRE1α phosphorylation by SR11302 **(*Figure 3D right panel*)**, however, may suggest a reciprocal regulation between AP-1 and IRE1α activation in myocardial I/R injury.

### sEH in global I/R-induced cardiac dysfunction: regulation by IRE1α-JNK-c-Jun/AP-1 signal axis

**Figure 4:**
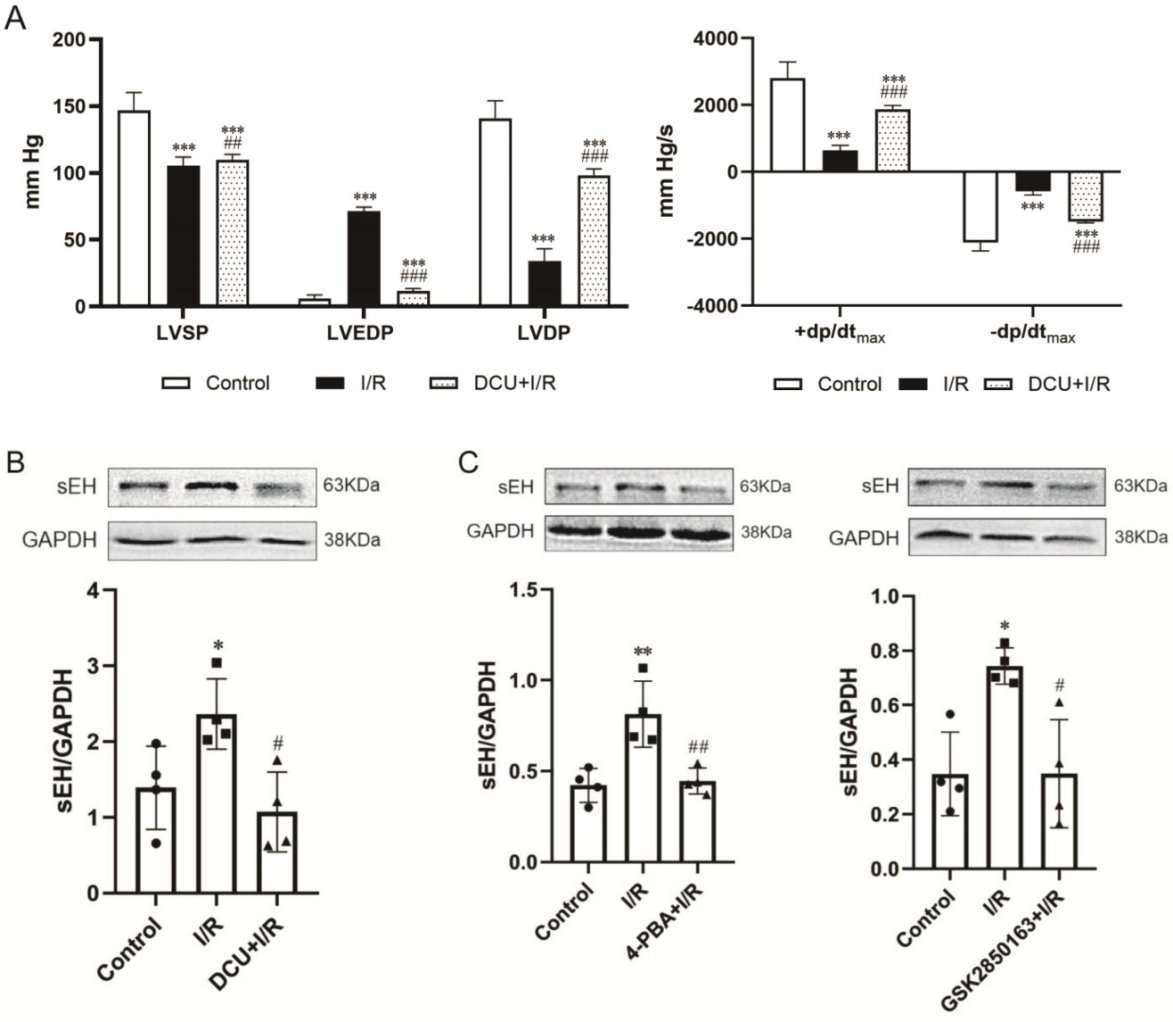

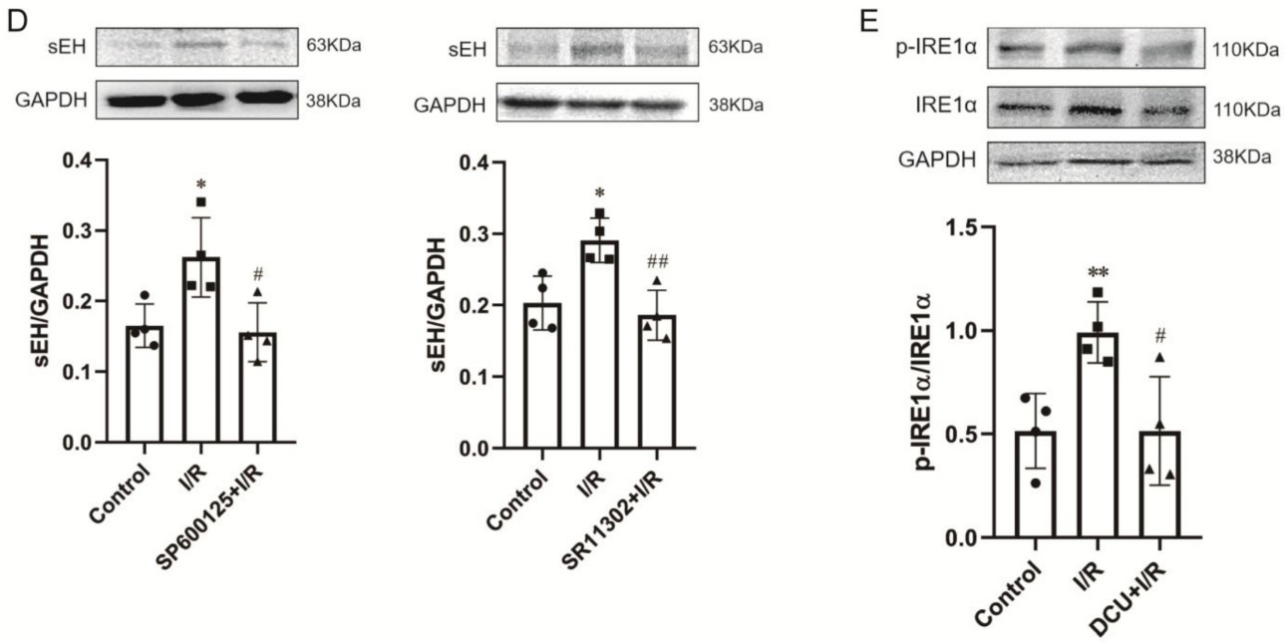
** sEH acts downstream of IRE1α-JNK-c-Jun/AP-1 in mediating global I/R-induced myocardial dysfunction. (A)** The sEH inhibitor DCU ameliorated systolic and diastolic dysfunction of I/R-injured heart (n=8). **(B)** I/R-induced upregulation of sEH was inhibited by DCU perfusion prior to ischemia (n=4). **(C)** Perfusion of the heart with 4-PBA or GSK2850136 to inhibit ER stress or the IRE1α branch of ER stress downregulated the expression of sEH in hearts subjected to I/R (n=4). **(D)** Both JNK and AP-1 inhibitors, SP600125 and SR11302, inhibited I/R-induced upregulation of sEH (n=4). **(E)** Inhibition of sEH by DCU attenuated I/R-induced phosphorylation of IRE1α (n=4). ^*^p<0.05, ^**^p<0.01, ^***^p<0.001 *vs.* Control; ^#^p<0.05, ^##^p<0.01, ^###^p<0.001 *vs.* I/R.

Perfusion with DCU, a potent sEH inhibitor, before I/R significantly improved the cardiac function with decreased LVEDP (11.7±1.8 vs. 71.4±3.0 mmHg, p<0.001) and increased LVSP (109.9±4.1 *vs.* 105.5±6.4 mmHg, p<0.05), LVDP (98.2±4.9 vs*.* 34.1±9.2 mmHg, p<0.001), +dp/dt_max_ (1869.8±121.9 vs*.* 651.7±142.1 mmHg/s, p<0.001), and -dp/dt_max_ (-1492.3±30.8 vs*.* -580.0±109.6 mmHg/s, p<0.001)** (*Figure 4A*)**. The protein expression of sEH was upregulated in the I/R-injured hearts, which could be downregulated by DCU treatment **(*Figure 4B*)**. The downregulation of sEH expression was also observed in I/R hearts perfused with the ER stress inhibitor 4-PBA and GSK2850163 **(*Figure 4C*)** as well as the JNK and AP-1 inhibitors SP600125 and SR11302 **(*Figure 4D*)**, suggesting the mediatory role of IRE1α/JNK/c-Jun signaling in I/R-induced sEH upregulation. Additionally, the upregulation of p-IRE1α in I/R hearts was suppressed by DCU pretreatment **(*Figure 4E*)**. These results might indicate an interplay between ER stress and sEH activation in myocardial I/R injury.

The baseline hemodynamic variables of the control, I/R group, and I/R groups pretreated with different inhibitors are listed in ***Table 1*,** showing no differences among groups. Comparisons of the protective effect of inhibitors on postischemic cardiac function are shown in ***Table 2***.

### Role of IRE1α-JNK-c-Jun/AP-1-sEH signal axis in global I/R-induced coronary endothelial dysfunction

**Figure 5:**
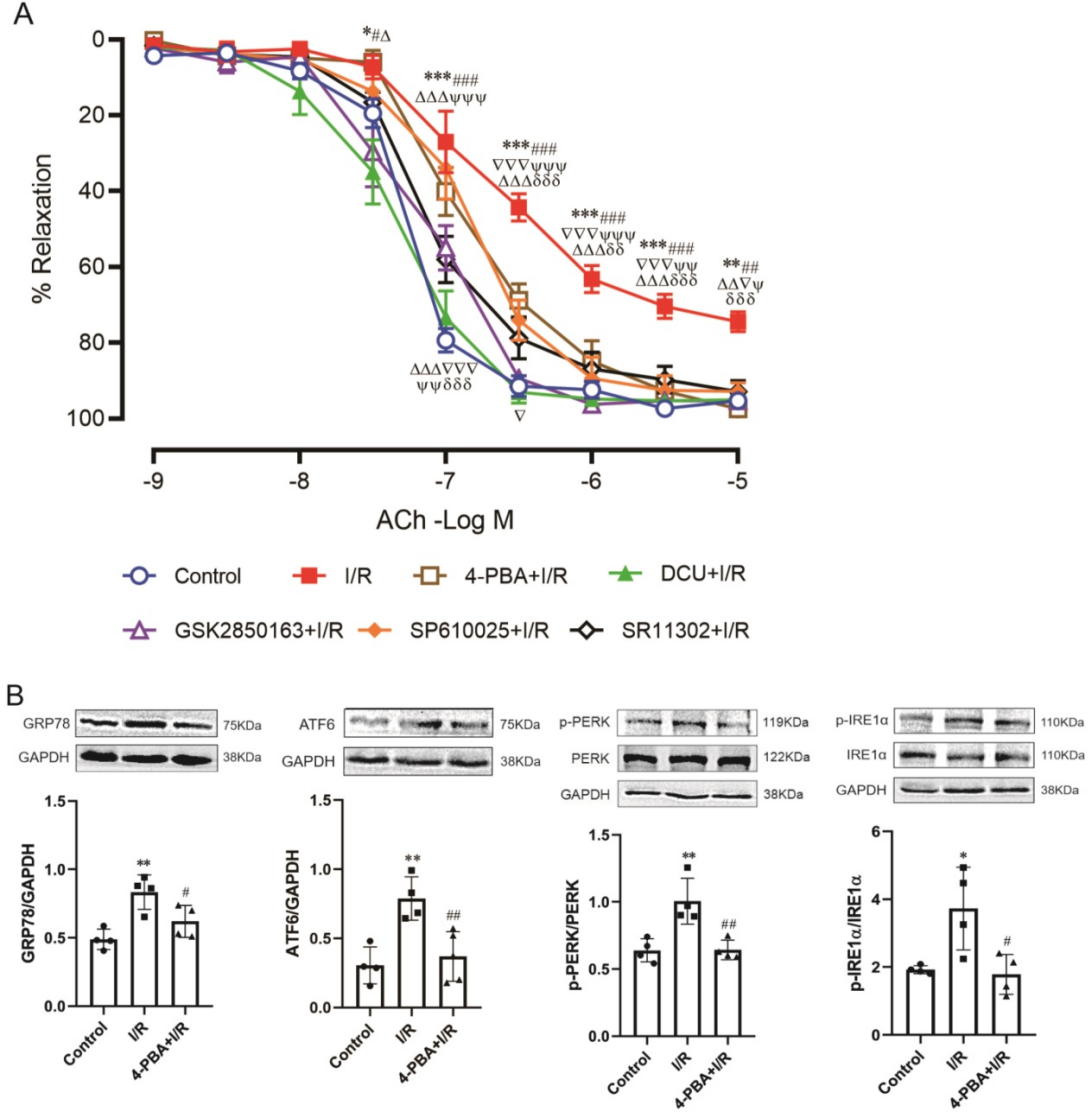

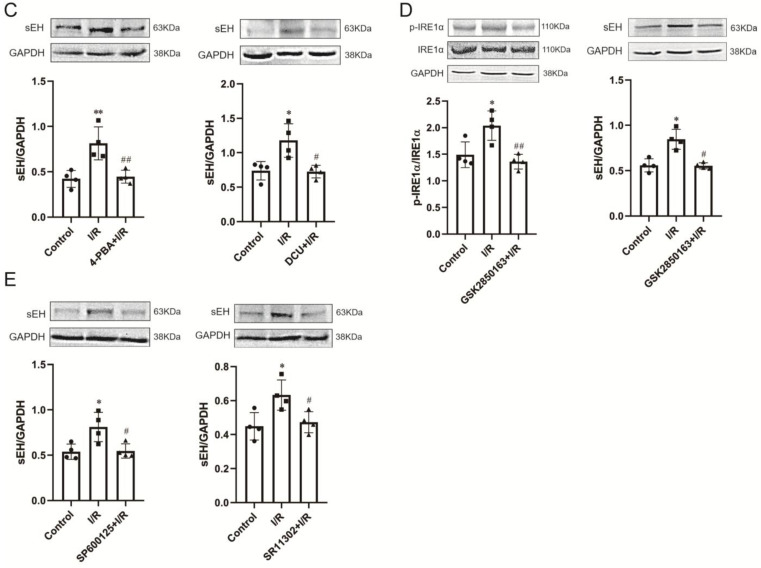
** Role and linkage of ER stress, JNK, c-Jun/AP-1, and sEH in global ischemia/reperfusion (I/R)-induced coronary endothelial dysfunction. (A)** Inhibition of ER stress, IRE1α, JNK, AP-1, or sEH improved endothelial dilator function of coronary arteries in heart subjected to I/R (n=6). ^*^p<0.05,^ ***^p<0.001 *vs.* Control; ^#^p<0.05, ^##^p<0.01, ^###^p<0.001 *vs.* DCU+I/R; ^Δ^p<0.05, ^ΔΔ^p<0.01, ^ΔΔΔ^p<0.001 *vs.* GSK2850163+I/R; ^▽^p<0.05, ^▽▽▽^p<0.001 *vs.* SP600125+I/R; ^Ψ^p<0.05, ^ΨΨ^p<0.01, ^ΨΨΨ^p<0.001 *vs.* SR11302+I/R; ^δδ^p<0.01, ^δδδ^p<0.001 *vs.* 4-PBA+I/R. **(B-E)** Western-blot showing the link among ER stress, JNK, c-Jun/AP-1, and sEH in coronary artery. I/R-induced expression/activation of ER stress molecules in the coronary artery was effectively inhibited by 4-PBA (**B**, n=4). The ER stress inhibitor 4-PBA downregulated I/R-induced sEH expression and the inhibitory effect was comparable to that of the sEH inhibitor DCU (**C**, n=4). Specifically inhibiting the activation of the IRE1α branch of ER stress with GSK2850163 prevented I/R-induced upregulation of sEH (**D**, n=4). Both JNK and AP-1 inhibitors SP600125 and SR11302 lowered I/R-induced increase of sEH in the coronary artery (**E**, n=4). ^*^p<0.05, ^**^p<0.01 *vs.* Control. ^#^p<0.05, ^##^p<0.01 *vs.* I/R.

The endothelium-dependent relaxation of coronary arteries was attenuated after 30-min ischemia and 60-min reperfusion. Both the magnitude of relaxant response (p<0.001 vs*.* control, two-way repeated measures) and the sensitivity to acetylcholine (EC_50_: -6.21±0.19 vs*.* -7.25±0.07 LogM, p<0.001) were decreased. Perfusion of the heart with 4-PBA before ischemia significantly ameliorated I/R-induced impairment of endothelial function (p<0.01 vs*.* I/R, two-way repeated measures) and functional restoration could also be achieved by GSK2850163 (p>0.05 vs*.* control, two-way repeated measures), which suggested the significance of the IRE1α branch of ER stress in I/R-induced endothelial dysfunction of coronary arteries. Inhibition of JNK and AP-1 with SP610025 and SR11302 also showed protective effect on acetylcholine-induced relaxation in coronary arteries. Moreover, in hearts perfused with DCU, the relaxant response of coronary arteries to acetylcholine was restored** (*Figure 5A*)**. These results collectively suggested the role of ER stress, JNK/c-Jun, and sEH in I/R-induced endothelial dysfunction. ***Table 3*** shows EC_50_ values and maximal relaxant response to acetylcholine of the coronary artery in each group.

Western-blot analysis further revealed the link among ER stress, JNK, c-Jun/AP-1, and sEH in I/R-induced endothelial dysfunction. As in myocardium, the expression of ER stress molecules was significantly increased in coronary arteries after I/R. Pretreatment with 4-PBA inhibited ER stress and downregulated sEH protein expression. Moreover, the inhibitory effect of 4-PBA on sEH expression was comparable to that of the sEH inhibitor DCU **(*Figure 5B&C*)**. Specifically inhibiting the activation of IRE1α branch of ER stress with GSK2850163 prevented I/R-induced upregulation of sEH as well **(*Figure 5D*)**. The I/R-induced increase of sEH in coronary arteries was also lowered in hearts perfused with the JNK and AP-1 inhibitors SP600125 and SR11302 **(*Figure 5E*)**. These data indicated that sEH functions as a downstream molecule of IRE1α, JNK, and c-Jun/AP-1 in the impairment of endothelial function in I/R condition.

## Discussion

Although previous studies have suggested that ER stress, JNK, and sEH participate in myocardial I/R injury, it remains unknown whether they are linked together to mediate I/R damage. In the present study, we revealed a link between ER stress and sEH dysregulation in global myocardial I/R injury with the finding of IRE1α-JNK-c-Jun/AP-1-sEH signaling axis. Our data demonstrated that activation of IRE1α mediates I/R-triggered upregulation of sEH via JNK-c-Jun/AP-1 pathway and this mechanism contributes to postischemic impairments of contractile/relaxant function of myocardium and endothelial function of the coronary artery. The identification of this mechanistic link between ER stress and sEH activation provided new insight into cardiac dysfunction following global myocardial I/R insult.

The Langendorff perfused heart is a physiologically relevant and controllable model that has been widely adopted for studies of global myocardial I/R injury. Comparing to most of the previous studies that focused on either myocardial function or coronary function, this study looked into both aspects. Our data suggested that ER stress, in particular the IRE1 branch of ER stress-mediated sEH upregulation is a common feature shared by myocardium and coronary artery in response to I/R insult. Perfusion of the heart with ER stress inhibitor or sEH inhibitor prior to I/R insult effectively ameliorated cardiac systolic and diastolic dysfunction and improved the dilator function of coronary artery. ER stress inhibitors showed a significant downregulating effect on I/R-induced sEH expression in both myocardium and coronary artery. We previously reported the role of ER stress in angiotensin II-induced sEH upregulation in coronary endothelial cells 12, the present study added another piece of evidence supporting the significance of ER stress-sEH signaling in cardiovascular pathology.

In this study, we also observed that pretreatment with the sEH inhibitor DCU suppresses the IRE1α activation in myocardium, as shown by decreased phosphorylation of IRE1α, which may imply an interplay between ER stress and sEH. Indeed, there were studies suggesting that sEH is a modulator of ER stress. For example, sEH deficiency/inhibition was found to significantly attenuate ER stress in the liver and adipose tissue in animals fed with high-fat diet 27. sEH inhibitor alleviated high glucose-induced mitochondrial dysfunction and ER stress in human proximal tubular epithelial cells and this mechanism was further proved to be responsible for its renal protective effect in db/db mice 28. With the finding of reciprocal regulation between sEH and IRE1α in this study, we assumed that a positive feedback between ER stress and sEH is involved in I/R-induced myocardial injury. Further in-depth studies using both cell and animal I/R models are warranted to validate this postulation.

The mediatory role of JNK-c-Jun/AP-1 in sEH upregulation promoted by IRE1 activation was supported by the results of western blot experiments. Perfusion of the heart with JNK or AP-1 inhibitors prior to I/R downregulated sEH expression, and the I/R-induced activation of JNK and c-Jun (shown by increased phosphorylation) was suppressed by the IRE1α inhibitor. Moreover, we observed that in I/R-injured heart, c-Jun phosphorylation at both Ser63 and Ser73 was significantly attenuated by the JNK inhibitor whereas inhibition of AP-1 did not modulate the phosphorylation of JNK, which suggested that c-Jun/AP-1 acts downstream of JNK to increase sEH expression in response to the activation of the IRE1α branch of ER stress. The finding of IRE1α-initiated JNK activation was consistent with previous studies 15, 16 and elucidation of the c-Jun/AP-1 mediation in IRE1α-driven sEH upregulation furthered our understanding of the role of JNK activation in myocardial I/R injury.

The inhibition of JNK and AP-1 activation was associated with an improvement in postischemic cardiac function and coronary dilator function. It seems that JNK and AP-1 may differ in their importance in systolic and diastolic function of the heart. Our data showed that inhibition of JNK protects systolic function better than diastolic function in heart subjected to I/R while AP-1 inhibition confers better protection on the postischemic diastolic function. Therefore, it is very likely that use of JNK inhibitor in conjunction with AP-1 inhibitor may yield a synergistic protective effect on myocardial function against I/R injury, though this needs to be confirmed through further experiments.

Considering that inappropriate coronary blood flow response may lead to deficient myocardial perfusion, we examined coronary dilator function in addition to myocardial contractility/relaxation in this study to more comprehensively understand how I/R affects cardiac function. Our finding of the impaired endothelium-dependent relaxation supported the notion that endothelial dysfunction of coronary artery is an important factor contributing to postischemic cardiac malperformance. The activation of IRE1α-JNK-c-Jun/AP-1/sEH signaling in coronary artery in response to I/R, same as observed in myocardium, suggested that this axis may present a promising target for prevention and treatment of myocardial I/R injury.

There are some limitations of this study that need to be addressed. Firstly, although we demonstrated that IRE1α activation in response to global myocardial I/R insult promotes sEH expression in myocardium and coronary artery, whether the other two UPR branches, PERK and ATF6, are also involved in the regulation of sEH under I/R condition was not investigated, which may be worth studying to further enrich our understanding of the association between ER stress and sEH changes in I/R injury. Secondly, we suggested a mutual regulation between IRE1α activation and sEH expression, however, no further efforts were made to understand how sEH modulates IRE1α. It might be possible that certain isoforms of EETs take part in the modulation though this needs to be demonstrated in future studies. Thirdly, we used the *ex vivo* Langendorff model to mimic global myocardial I/R in cardiac surgery and demonstrated the effectiveness of different drugs in ameliorating I/R injury. Given that in cardiac surgery with cardiopulmonary bypass, the heart is rapidly arrested with cardioplegia, future studies with inclusion of the cardioplegia procedure, either by administration of cardioplegia following the drug perfusion or administration of the drug-supplemented cardioplegia, could even better simulate the clinical condition. Moreover, extrapolating the results to regional myocardial I/R needs to be cautious, which should be verified in future studies using *in vivo* animal model.

In conclusion, this study revealed a signaling linkage between ER stress and sEH regulation in cardiac I/R injury. ER stress upregulates sEH expression through IRE1α-JNK-c-Jun/AP-1 signaling axis, leading to myocardial and coronary dysfunction. This study provided new insight into the mechanisms underlying postischemic myocardial injury and may suggest the potential of targeting the IRE1α-JNK-c-Jun/AP-1-sEH signaling axis for cardioprotection.

## Supplementary Material

Supplementary materials and methods, figure.

## Figures and Tables

**Table 1:** Baseline hemodynamic variables of the study groups

Group	LVSP (mmHg)	LVEDP (mmHg)	LVDP (mmHg)	+dp/dt_max_ (mmHg/s)	-dp/dt_max_ (mmHg/s)
Control	132.2±4.1	4.3±2.7	127.9±5.0	2707.0±74.3	-2075.2±184.5
I/R	135.5±6.0	6.9±3.1	128.6±7.1	2800.9±264.9	-1921.5±335.4
4-PBA+I/R	133.1±5.3	5.1±2.9	128.1±2.7	2608.0±119.6	-2058.5±126.2
GSK2850163+I/R	132.2±5.4	5.1±3.6	127.1±6.2	2963.7±128.8	-2074.1±419.8
SP610025+I/R	133.8±12.0	6.7±3.1	127.1±11.6	3093.2±376.4	-2133.6±199.2
SR11302+I/R	135.1±4.8	4.6±2.7	130.5±4.2	3044.9±473.4	-2051.3±322.3
DCU+I/R	135.2±6.4	5.4±3.4	129.8±7.0	2588.7±483.2	-1989.5±144.2

n=8 in each group. **LVSP**: left ventricular systolic pressure; **LVEDP**: left ventricular end-diastolic pressure; **LVDP**: left ventricular developed pressure; **+dp/dt_max_** and** -dp/dt_max_** = maximal velocity of contraction and relaxation of the left ventricle.

**Table 2:** Comparisons of the postischemic hemodynamic performance among groups

Group	LVSP (mmHg)	LVEDP (mmHg)	LVDP (mmHg)	+dp/dt_max_ (mmHg/s)	-dp/dt_max_ (mmHg/s)
Control	146.9±13.4	6.0±2.7	140.9±13.1	2806.6±480.6	-2118.0±244.9
I/R	105.5±6.4^***^	71.4±3.0^***^	34.1±9.2^***^	651.7±142.1^***^	-580.0±109.6^***^
4-PBA+I/R	119.5±15.6^***##^	21.2±4.2^***###^	98.3±12.0^***###^	2166.7±208.4^***###^	-1350.9±99.8^***###^
GSK2850163+I/R	113.4±10.9^***##^	37.1±3.1^***###∇∇∇^	76.3±13.9^***###∇∇∇^	1586.5±263.3^***###∇∇∇^	-1127.7±159.9^***###∇∇∇^
SP610025+I/R	113.9±5.6^***##^	73.4±4.9^***∇∇∇△△△^	40.5±3.3^***#∇∇∇△△△^	970.1±89.8^***##∇∇∇△△△^	-616.9±81.5^∇∇∇△△△^
SR11302+I/R	97.9±7.5^***##∇△◇◇^	52.7±8.6^***###∇∇∇△△◇◇◇^	45.2±9.8^***#∇∇∇△△△◇^	1231.5±196.6^***##∇∇∇△◇◇◇^	-658.3±68.9^***#∇∇∇△△△◇◇◇^
DCU+I/R	109.9±4.1^***##☆^	11.7±1.8^***###∇∇∇△△△☆☆☆^	98.2±4.9^***###△△△☆☆☆^	1869.8±121.9^***###∇∇∇△△☆☆☆^	-1492.3±30.8^***###∇∇∇△△△☆☆☆^

n=8 in each group. ^***^p<0.001 *vs.* Control; ^#^*p*<0.05, ^##^*p*<0.01, ^###^*p*<0.001 *vs.* I/R; ^∇^*p*<0.05, ^∇∇∇^*p*<0.001 *vs.* 4-PBA+I/R; ^△^*p*<0.05, ^△△^*p*<0.01, ^△△△^*p*<0.001 *vs.* GSK2850163+I/R;^ ◇^*p*<0.05, ^◇◇^*p*<0.01, ^◇◇◇^*p*<0.001 *vs.* SP610025+I/R; ^☆^*p*<0.05, ^☆☆^*p*<0.01, ^☆☆☆^*p*<0.001 *vs.* SR11302+I/R. **LVSP**: left ventricular systolic pressure; **LVEDP**: left ventricular end-diastolic pressure; **LVDP**: left ventricular developed pressure; **+dp/dt_max_** and** -dp/dt_max_** = maximal velocity of contraction and relaxation of the left ventricle.

**Table 3:** EC_50_ values and maximal relaxant response of coronary arteries to acetylcholine

Group	EC_50_ (LogM)	Rmax (%)
Control	-7.25±0.07	95.3±4.2
I/R	-6.21±0.19^***^	74.5±6.2^**^
4-PBA+I/R	-6.75±0.20^**##^	97.4±9.4^###^
GSK2850163+I/R	-7.15±0.22^###^	95.3±4.0^##^
SP610025+I/R	-6.80±0.27^*###^	92.8±5.5^#^
SR11302+I/R	-6.97±0.22^###^	92.9±7.0^#^
DCU+I/R	-7.33±0.27^###∇∇∇^	95.0±4.1^##^

n=6 in each group. ^*^p<0.05, ^**^p<0.01, ^***^p<0.001 *vs.* Control; ^#^p<0.05, ^##^p<0.01, ^###^p<0.001* vs.* I/R. ^∇∇∇^*p*<0.001 *vs.* 4-PBA+I/R. **4-PBA**: ER stress inhibitor; **GSK2850163**: IRE1α inhibitor; **SP610025**: JNK inhibitor; **SR11302**: AP-1 inhibitor; **DCU**: sEH inhibitor.

## References

[1] Oakes SA, Papa FR (2015). The role of endoplasmic reticulum stress in human pathology. Annu Rev Pathol.

[2] Zhu H, Zhou H (2021). Novel insight into the role of endoplasmic reticulum stress in the pathogenesis of myocardial ischemia-reperfusion injury. Oxid Med Cell Longev.

[3] Yu L, Li B, Zhang M, Jin Z, Duan W, Zhao G (2016). Melatonin reduces PERK-eIF2alpha-ATF4-mediated endoplasmic reticulum stress during myocardial ischemia-reperfusion injury: role of RISK and SAFE pathways interaction. Apoptosis.

[4] Jin JK, Blackwood EA, Azizi K, Thuerauf DJ, Fahem AG, Hofmann C (2017). ATF6 decreases myocardial ischemia/reperfusion damage and links ER stress and oxidative stress signaling pathways in the heart. Circ Res.

[5] Harris TR, Hammock BD (2013). Soluble epoxide hydrolase: gene structure, expression and deletion. Gene.

[6] Morisseau C, Hammock BD (2013). Impact of soluble epoxide hydrolase and epoxyeicosanoids on human health. Ann Rev Pharmacol Toxicol.

[7] Katragadda D, Batchu SN, Cho WJ, Chaudhary KR, Falck JR, Seubert JM (2009). Epoxyeicosatrienoic acids limit damage to mitochondrial function following stress in cardiac cells. J Mol Cell Cardiol.

[8] Motoki A, Merkel MJ, Packwood WH, Cao Z, Liu L, Iliff J (2008). Soluble epoxide hydrolase inhibition and gene deletion are protective against myocardial ischemia-reperfusion injury *in vivo*. Am J Physiol Heart Circ Physiol.

[9] Islam O, Patil P, Goswami SK, Razdan R, Inamdar MN, Rizwan M (2017). Inhibitors of soluble epoxide hydrolase minimize ischemia-reperfusion-induced cardiac damage in normal, hypertensive, and diabetic rats. Cardiovasc Ther.

[10] Ding Y, Li Y, Zhang X, He J, Lu D, Fang X (2017). Soluble epoxide hydrolase activation by S-nitrosation contributes to cardiac ischemia-reperfusion injury. J Mol Cell Cardiol.

[11] Ai D, Fu Y, Guo D, Tanaka H, Wang N, Tang C (2007). Angiotensin II up-regulates soluble epoxide hydrolase in vascular endothelium *in vitro* and *in vivo*. Proc Natl Acad Sci U S A.

[12] Mak SK, Yu CM, Sun WT, He GW, Liu XC, Yang Q (2017). Tetramethylpyrazine suppresses angiotensin II-induced soluble epoxide hydrolase expression in coronary endothelium via anti-ER stress mechanism. Toxicol Appl Pharmacol.

[13] Shvedova M, Anfinogenova Y, Atochina-Vasserman EN, Schepetkin IA, Atochin DN (2018). c-Jun N-terminal kinases (JNKs) in myocardial and cerebral ischemia/reperfusion injury. Front Pharmacol.

[14] Urano F, Wang X, Bertolotti A, Zhang Y, Chung P, Harding HP (2000). Coupling of stress in the ER to activation of JNK protein kinases by transmembrane protein kinase IRE1. Science.

[15] Liang Y, Liang L, Liu Z, Wang Y, Dong X, Qu L (2020). Inhibition of IRE1/JNK pathway in HK-2 cells subjected to hypoxia-reoxygenation attenuates mesangial cells-derived extracellular matrix production. J Cell Mol Med.

[16] Wu T, Jiang N, Ji Z, Shi G (2019). The IRE1 signaling pathway is involved in the protective effect of low-dose LPS on myocardial ischemia-reperfusion injury. Life Sci.

[17] Wang XC, Sun WT, Yu CM, Pun SH, Underwood MJ, He GW (2015). ER stress mediates homocysteine-induced endothelial dysfunction: modulation of IKCa and SKCa channels. Atherosclerosis.

[18] Concha NO, Smallwood A, Bonnette W, Totoritis R, Zhang G, Federowicz K (2015). Long-range inhibitor-induced conformational regulation of human IRE1alpha endoribonuclease activity. Mol Pharmaco.

[19] Bennett BL, Sasaki DT, Murray BW, O'Leary EC, Sakata ST, Xu W (2001). SP600125, an anthrapyrazolone inhibitor of Jun N-terminal kinase. Proc Natl Acad Sci U S A.

[20] Guo C, Wang SL, Xu ST, Wang JG, Song GH (2015). SP600125 reduces lipopolysaccharide-induced apoptosis and restores the early-stage differentiation of osteoblasts inhibited by LPS through the MAPK pathway in MC3T3-E1 cells. Int J Mol Med.

[21] Xie L, Feng H, Li S, Meng G, Liu S, Tang X (2016). SIRT3 Mediates the antioxidant effect of hydrogen sulfide in endothelial cells. Antioxid Redox Signal.

[22] Maron BA, Oldham WM, Chan SY, Vargas SO, Arons E, Zhang YY (2014). Upregulation of steroidogenic acute regulatory protein by hypoxia stimulates aldosterone synthesis in pulmonary artery endothelial cells to promote pulmonary vascular fibrosis. Circulation.

[23] Fang X, Hu S, Xu B, Snyder GD, Harmon S, Yao J (2006). 14,15-Dihydroxyeicosatrienoic acid activates peroxisome proliferator-activated receptor-alpha. Am J Physiol Heart Circ Physiol.

[24] Jiang H, Zhu AG, Mamczur M, Morisseau C, Hammock BD, Falck JR (2008). Hydrolysis of cis- and trans-epoxyeicosatrienoic acids by rat red blood cells. J Pharmacol Exp Ther.

[25] Yang Q, Hohimer AR, Giraud GD, Van Winkle DM, Underwood MJ, He GW (2008). Effect of fetal anaemia on myocardial ischaemia-reperfusion injury and coronary vasoreactivity in adult sheep. Acta Physiol (Oxf).

[26] Yang Q, Xue HM, Wong WT, Tian XY, Huang Y, Tsui SK (2011). AVE3085, an enhancer of endothelial nitric oxide synthase, restores endothelial function and reduces blood pressure in spontaneously hypertensive rats. Br J Pharmacol.

[27] Bettaieb A, Nagata N, AbouBechara D, Chahed S, Morisseau C, Hammock BD (2013). Soluble epoxide hydrolase deficiency or inhibition attenuates diet-induced endoplasmic reticulum stress in liver and adipose tissue. J Biol Chem.

[28] Jiang XS, Xiang XY, Chen XM, He JL, Liu T, Gan H (2020). Inhibition of soluble epoxide hydrolase attenuates renal tubular mitochondrial dysfunction and ER stress by restoring autophagic flux in diabetic nephropathy. Cell Death Dis.

