# Supplementary material for: Targeting IRE1α-JNK-c-Jun/AP-1-sEH Signaling Pathway Improves Myocardial and Coronary Endothelial Function Following Global Myocardial Ischemia/Reperfusion
Source: Int J Med Sci. 2022 Aug 15;19(9):1460–72. doi: 10.7150/ijms.74533 (PMC9413556; doi:10.7150/ijms.74533)
Supplement: Supplementary file 1 — Supplementary materials and methods, figure. [file ijmsv19p1460s1.pdf]

Supplementary Materials

Materials & Methods

Chemical and Reagents

ER stress inhibitor 4-phenylbutyric acid (4-PBA, P132032) was purchased from Aladdin (Shanghai, China). IRE1α inhibitor GSK2850163 (HY-U00459), JNK inhibitor SP600125 (HY-12041), and phenylephrine (HY-B0769) were purchased from MedChemExpress (Shanghai, China). AP-1 inhibitor SR11302 (16338) was from Cayman Chemical (Michigan, USA) and sEH inhibitor N,N'-Dicyclohexylurea (DCU, 324810) from Calbiochem (Darmstadt, Germany). Acetylcholine (ACh, A6625) was purchased from Sigma-Aldrich.

Krebs-Henseleit buffer (KHB) (in mmol/L: NaCl 118.5, KCl 4.7, $NaHCO_3$ 25, KH_2PO_4 1.2, $MgSO_4$ 1.2, glucose 11, and $CaCl_2$ 1.5) was freshly prepared before the experiment.

Western blotting

The cardiac tissues and coronary arteries were homogenated and extracted for protein with RIPA lysis buffer containing protease and phosphatase inhibitor cocktail (Thermo Fisher Scientific, Shanghai, China). The protein concentration was determined by a bicinchoninic acid assay kit (Beyotime Biotechnology, China). Protein extracts were separated by sodium dodecyl sulfate polyacrylamide gel (SDS-PAGE) electrophoresis then electrotransferred to a polyvinylidenedifluoride (PVDF) membrane (Thermo Fisher Scientific) for detection of the protein of interest. After blocking with 5% bovine serum albumin for 1h at room temperature, the PVDF membrane was incubated with primary antibodies overnight at 4°C against PERK (1:500, Cat#3192, RRID:AB_2095847, Cell Signaling Technology), phosphorylated (Thr980) PERK (p-PERK, 1:500, Cat#bs-3330R, Bioss), IRE1α (IRE1α, 1:1000, Cat#ab37073, RRID:AB_775780, Abcam), phosphorylated (Ser724) IRE1α (1:500, Cat#ab48187, RRID:AB_873899, Abcam), ATF6 (1:500, Cat#ab203119, RRID:AB_2650448, Abcam), GRP78 (1:2000, Cat#ab21685, RRID:AB_2119834, Abcam), phospho-JNK1+JNK2 (Thr183+Tyr185) (1:500, Cat#ab4821, Abcam), JNK (1:500, Cat#9258, RRID:AB_2141027, Cell Signaling Technology), phospho-c-Jun (Ser63) (1:500,

Cat#ab32385, RRID:AB_726900, Abcam), phospho-c-Jun (Ser73) (1:500, Cat#3270, RRID:AB_2129575, Cell Signaling Technology), c-Jun (1:1000, Cat#9165, RRID:AB_2130165, Cell Signaling Technology), and sEH (1:1000, Cat#E-AB-60489, Elabscience). The membrane was then probed with horseradish peroxidase (HRP)-conjugated secondary antibody (1:1000, Cat#7076, Cat#7074, Cell Signaling Technology) for 1h at room temperature. GAPDH (1:1000, Cat#10494-1-AP, RRID:AB_2263076, Proteintech) was used as internal loading control. The protein bands were visualized by ECL reagent (Beyotime Biotechnology, China) and analyzed by Quantity one imaging system version4.6.6 (Bio-Rad, Colombia, US).

Figure S1.

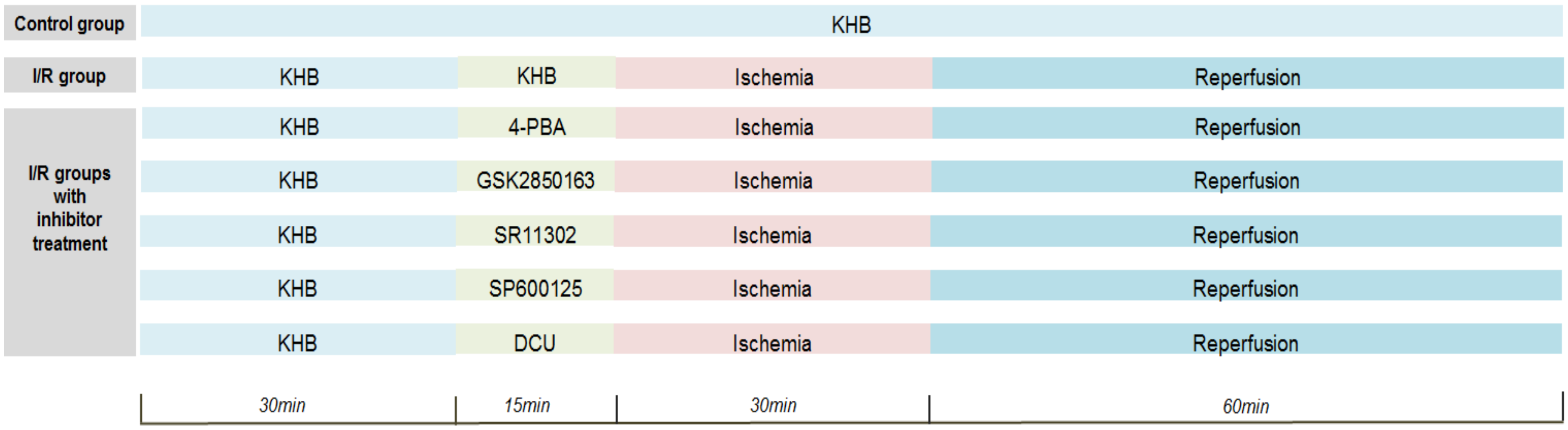

Figure S1. Schematic illustration of experimental protocols. The freshly isolated rat heart was mounted on a Langendorff apparatus. In the control group, the heart was perfused with warm Krebs-Henseleit buffer (KHB) at a constant hydrostatic pressure of 70-80mmHg throughout the experiment. In ischemia/reperfusion (I/R) groups without or with inhibitor pretreatment, the heart was stabilized with KHB perfusion for 30-min then perfused for another 15 min with KHB or KHB containing one of the following inhibitors including the ER stress inhibitor 4-PBA, the IRE1α inhibitor GSK2850163, the JNK inhibitor SP600125, the AP-1 inhibitor SR11302, and the sEH inhibitor DCU. The hearts were then subjected to 30-min ischemia and 60-min reperfusion.
